# Dr. Comfort Edson Peck

**Published:** 1915-10

**Authors:** 


**Dr. Comfort Edson Peck,** Buffalo 1873, Bellevue 1879, died at his home in Highland Park, Ill., September 2, aged 69. He was vice-president of the Bowman Dairy Co. for 30 years.

